# Efficient and Stable CdSe/CdS/ZnS Quantum Rods-in-Matrix Assembly for White LED Application

**DOI:** 10.3390/nano10020317

**Published:** 2020-02-12

**Authors:** Yujuan Chen, Weishuo Xing, Yixuan Liu, Xinsu Zhang, Yangyang Xie, Chongyu Shen, Jay Guoxu Liu, Chong Geng, Shu Xu

**Affiliations:** 1 Tianjin Key Laboratory of Electronic Materials and Devices, School of Electronics and Information Engineering, Hebei University of Technology, 5340 Xiping Road, Tianjin 300401, China; dundun_chen@foxmail.com (Y.C.); xingweishuo54@163.com (W.X.); lyxlyx2016@163.com (Y.L.); cmjq18809@foxmail.com (X.Z.); xyyhebut@163.com (Y.X.); 2 SHINEON Co., LTD., Building 3, No. 58 Jinghai Road, BDA, Beijing 100176, China; chongyushen@shineon.cn (C.S.); jayliu@shineon.cn (J.G.L.)

**Keywords:** quantum rod, core-shell, assembly, light-emitting diodes, stability

## Abstract

CdSe/CdS core-shell quantum rods (QRs) are a promising prospect in optoelectronic applications but usually have a relatively low quantum efficiency and stability. Here, we report on an efficient and stable CdSe/CdS/ZnS QRs-in-matrix assembly (QRAs) by growing and embedding CdSe/CdS QRs in ZnS matrices. Structural characterizations show that the CdSe/CdS QRs are encapsulated and interconnected by ZnS in the QRAs structure. The stable ZnS encapsulation renders the CdSe/CdS QRs high quantum efficiency (QE) up to 85%. The QRAs also present high photo- and thermal-stability and can preserve 93% of the initial QE at 100 °C. The QRAs powder presents a light degradation of only 2% under continuous excitation for 100 h, displaying profound potential in optoelectronic applications. White light-emitting diodes (WLEDs) are fabricated by packaging the QRAs powder as phosphor on top of blue GaN chip. The WLED shows high optical performance and light quality.

## 1. Introduction

CdSe/CdS dot-in-rod nanocrystals integrate the 0D confined quantum dots (QDs) within 1D quantum rods [1,2,3,4]. This heterostructure endows the CdSe/CdS quantum rods (QRs) with a larger Stokes shift, intense polarized emission, faster radiative decay, and intrinsic charge separation [5,6,7,8]. These unique optical and electrical features make CdSe/CdS QRs promising for optoelectronic applications such as light-harvesting [7], biosensing [4], visible lighting communication [9,10,11], and light-emitting diodes (LEDs) [12,13]. Conventional CdSe/CdS QDs present a large overlap between the absorption and emission spectra that induces strong concentration quenching of QDs and a thermal issue in optoelectronic devices [14,15,16,17,18]. For CdSe/CdS QRs, the CdS rod acts as an efficient absorber for light below 470 nm, and the generated excitons in CdS can transfer to the quantum confined CdSe core and promote emission at longer wavelengths [19,20]. This characteristic is important for lighting and display applications in which the mixed green and red emitting QDs and QRs are excited by blue LEDs [21,22,23]. Because of the significant extinction coefficient, the large volume of CdS strongly increases the blue absorbance and reduces the demand for the concentration of the emissive CdSe core [24,25]. As a result, concentration quenching is effectively inhibited.

In previous decades, considerable progress has been made in the synthesis and optical study of CdSe/CdS QRs [1,2,3,4,5,6,7,8,9]. However, the practical application of CdSe/CdS and CdSe/CdS/ZnS QRs is still limited because of their relatively low quantum efficiency (QE) and stability compared to the “giant” spherical CdSe/CdS/ZnS QDs [26,27]. This issue is mainly attributed to the surface defects on CdS, which trap the electrons and induce non-radiative recombination [28,29,30]. The spherical CdSe/CdS QDs usually possess a cubic structure, and the CdS surface can be passivated by overgrowing a thick ZnS shell [31,32]. However, the CdS rods have a hexagonal structure, and the lattice mismatch between hexagonal CdS and ZnS is rather large, so it is unfavorable to grow a thick ZnS shell on CdS QRs [33,34]. Although the efficiency of CdSe/CdS QRs has been improved by passivation of the surface with organic ligands and thin ZnS encapsulation, the thermal- and photo-stability of the CdSe/CdS/ZnS QRs remains unsatisfactory [35,36,37].

In this article, we report on the efficiency and stability enhancement of the CdSe/CdS QRs by forming a CdSe/CdS/ZnS QRs-in-matrix assembly (QRAs) structure. The QRAs were synthesized by growing ZnS on CdSe/CdS QRs at a low ligand concentration. The ZnS particles crosslink the CdSe/CdS QRs and fabricate a matrix involving the QRs and ZnS crosslinker. Steady-state and real-time photoluminescence characterizations were employed to investigate the optical properties of the QRAs under different conditions. White-light LEDs (WLEDs) based on QRAs were further fabricated to verify the high performance of the QRAs in practical optoelectronic applications.

## 2. Materials and Methods

### 2.1. Materials

Cadmium oxide (CdO, 325 mesh, 99.5%) and selenium powder (Se, 325 mesh, 99.5%) were purchased from Alfa Aesar (China) Chemicals Co., Ltd. Cadmium stearate (Cd(St)_2_) was purchased from Shanghai Debo Chemical Technology Co., Ltd. Octadecylphosphonic acid (ODPA, 98%) was purchased from EPSILON CHIMIE (Brittany, France). Hexylphosphonic acid (HPA, 98%) was purchased from IRRITANT. 1-Octadecene (ODE, >90%), zinc diethyldithiocarbamate (Zn(DDTC)_2_, >99%), hexyldecylamine (HDA, >95%), sulfur (S, 98.5%), and trioctylphosphine (TOP, 85%) were purchased from TCI (Shanghai) Development Co., Ltd. Trioctylphosphine oxide (TOPO, 98%) was purchased from Shanghai Macklin Biochemical Co., Ltd. Ethanol (AR) and dimethylbenzene were purchased from Tianjin Damao Chemical Reagent Co., Ltd. Silicone resin (Dow Corning-6662) was from Dow Corning. Lu_3_Al_5_O_12_:Ce (LuAG:Ce) phosphor was from Grirem Advanced Materials Co., Ltd. All chemicals were used directly without further purification unless otherwise stated. Table 1 shows the information of the purchased chemicals.

### 2.2. Synthesis of CdSe/CdS QRs and CdSe/CdS/ZnS QRAs

Synthesis of CdSe QDs: 3.156 g of TOPO (8 mmol), 0.273 g of ODPA (0.8 mmol), 0.065 g of CdO (0.5 mmol), and 10 mL of ODE were added into a three-necked flask. After vacuum degassing, the mixture was heated to 150 °C for 1 h under nitrogen. Then, the temperature was raised to 370 °C. TOP (2.5 mL) was injected after the solution turned from reddish to colorless at around 300 °C. After the formation of Cd-ODPA, a solution of 0.06 g of Se and 0.5 mL of TOP was injected quickly. After 30 s, the solution was cooled to room temperature. Approximately 10 mL of xylene and 20 mL of ethanol were added into the solution, and the solution was centrifuged at 4000 rpm for 10 min. The precipitate was collected, dried, and dispersed in TOP to obtain a TOP solution of CdSe QDs.

Synthesis of CdSe/CdS QRs: 0.065 g of CdO (0.5 mmol), 3.156 g of TOPO (8 mmol), 0.290 g of ODPA (0.85 mmol), 0.085 g of HPA (0.5 mmol), and 10 mL of ODE were added into a three-necked flask. After vacuum degassing for about 1 h at 150 °C, the mixture was heated to 350 °C under nitrogen. After the CdO powder was dissolved, TOP (2.5 mL) was injected into the mixture at about 300 °C and the solution became colorless. After reaching the desired temperature, a solution of 0.120 g of S and 2.5 mL of TOP and a mixed solution of 213 μL of CdSe QDs-TOP solution (0.4 mmol of QDs per liter of TOP) were injected rapidly. After stirring for 6 min, the reaction was terminated by ice bath; then, CdSe/CdS QRs were washed with 10 mL of xylene and 20 mL of ethanol.

Synthesis of CdSe/CdS/ZnS QRAs: After the synthesis, the QRs were purified and re-dissolved in ODE to form a 2.5 × 10^−5^ mol/L solution. Then, 0.2 mmol of Zn(DDTC)_2_ and 0.1 mL of HDA were added to the QRs-ODE solution. The mixture was heated to 150 °C under N_2_ and stirring was maintained for 10 min. Then, the solution was heated to 220 °C and stirring was maintained for 10 min. After the reaction, the solution was cooled down to room temperature and added to 2 mL of toluene and 10 mL of ethanol until the solution became turbid. The solution was then centrifuged at 4000 rpm for 5 min, and the precipitate was collected and dried to obtain the QRAs power.

### 2.3. Fabrication of WLEDs with QRs and QRAs

The experiment used a typical 2835 lead frame LED (2.8 mm × 3.5 mm × 0.65 mm) package. The experiment was carried out by mixing LuAG:Ce phosphor and CdSe/CdS/ZnS QRAs or CdSe/CdS QRs powder into silicone resin. Then, the mixture was deposited on top of the GaN LED chip in the LED frame through fluid dispensing. The fabricated LEDs were kept at 100 °C for 2 h and 150 °C for 2 h in air to cure the phosphor–silicone composite. The LEDs were soldered on PCB by a standard reflow soldering process to carry out the optical and stability tests.

### 2.4. Characterizations

Photoluminescence (PL) and stability tests were acquired on an Ideaoptics FX2000-EX PL-spectrometer. Transmission electron spectroscopy (TEM) and high-angle annular dark-field scanning transmission electron microscope (HAADF-STEM) micrographs was performed on a FEI TalosF200s transmission electron microscope operating at 100 kV. Scanning electron microscopy (SEM) experiments were performed on FEI Nova NanoSEM 450. Fourier transform infrared spectroscopy (FTIR) measurements were carried out on a Thermo-Nicolet iS50 FTIR-spectrometer. The quantum efficiency (QE) measurements were carried out on an Ocean Optics QEpro QY test system under 390 nm blue laser irradiation. The infrared thermal images were recorded on an Optris PI200. The luminous efficiency and optical power were recorded on an EVERFINE ATA-1000 LED automatic temperature control photoelectric analysis and measurement system. Ultraviolet visible (UV-Vis) absorption spectra were tested on a Persee T6 UV-Vis spectrometer. Thermal gravimetric (TG) analysis was performed on a Beijing lasting Scientific Instrument Factory HCT-3 Microcomputer Differential Thermal Balance. The luminous efficiency and optical power were recorded on an EVERFINE ATA-1000 LED automatic temperature control photoelectric analysis and measurement system. The fluorescent thermal quenching was tested on an EVERFINE EX-1000 test system.

## 3. Results and Discussion

### 3.1. Growth and Structure Characterization of CdSe/CdS/ZnS QRAs

CdSe/CdS QRs were synthesized by referring to the literature [37]. As mentioned above, the ZnS has a large lattice mismatch with hexagonal CdS. Hence, it usually forms a thin-layer encapsulation on the QR surface. According to literature reports, when there are plenty of ligands in a solution, overgrowth of thick ZnS will drive the destruction of the ZnS shelling on isolated CdSe/CdS QRs [33,34]. The ligands help to maintain the monodispersion of the QRs in solution; however, they are also involved into the resolve-growth of the surface during ZnS shelling and accelerate the surface reconstruction. With this understanding, we reduced the ligand concentration in the growth of ZnS on the surface of CdSe/CdS QRs, and Zn(DDTC)_2_ was employed as a single source ZnS precursor. The growth led to the formation of the assembly structure of CdSe/CdS QRs with ZnS encapsulation and interconnection.

**Figure 1:**
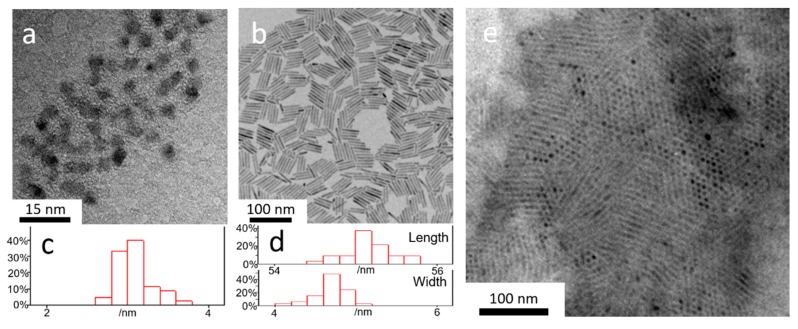
TEM images of (**a**) CdSe quantum dots (QDs); (**b**) CdSe/CdS quantum rods (QRs) and (**e**) CdSe/CdS/ZnS QRs-in-matrix assembly (QRAs) and the size distribution of the (**c**) QDs and (**d**) QRs.

Figure 1 shows the TEM images of the CdSe QDs core, CdSe/CdS QRs, and the CdSe/CdS/ZnS QRAs. The CdSe QDs present a quasi-spherical shape with a mean diameter of 3.1 nm. Figure 1b displays homogenously dispersed CdSe/CdS QRs with an average length of 55 nm and width of 4.7 nm, respectively. Figure 1c,d present the charts of the size distribution for the CdSe QDs and the CdSe/CdS QRs, which were measured from the TEM images. After the growth of ZnS, the CdSe/CdS QRs became closely packed and connected with each other to form an assembly structure, as shown in Figure 1e. The CdSe/CdS QRs in the assembly present a slightly increased mean diameter of 5.1 nm, suggesting the growth of the ZnS layer on the surface.

**Figure 2:**
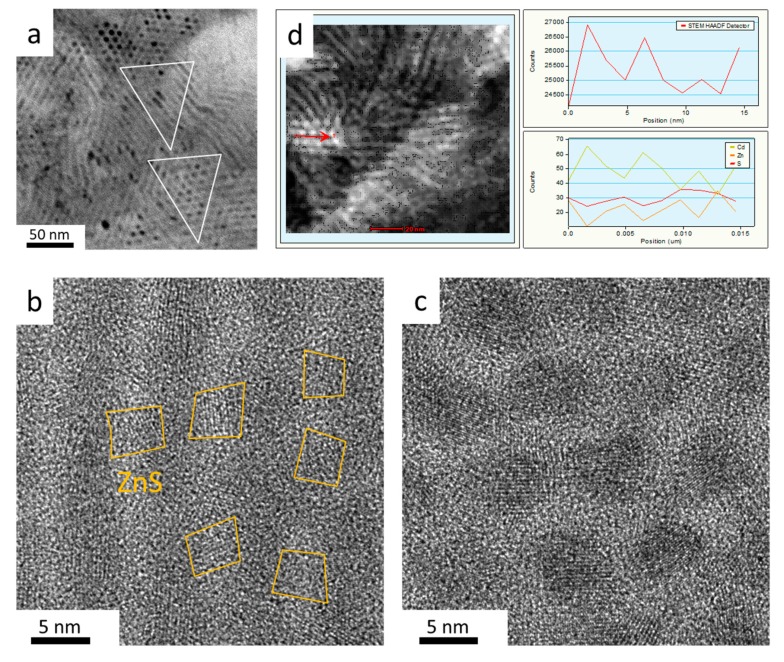
(**a**) TEM and HRTEM images of the side (**b**) and top (**c**) view of the CdSe/CdS/ZnS QRAs. (**d**) HAADF element analysis of the QRAs in the selected line.

The high resolution TEM (HRTEM) image of selected areas in Figure 2a shows the existence of ZnS between CdSe/CdS QRs (Figure 2a–c). Some of the ZnS particles are marked with the yellow frame in Figure 2b. This shows that the CdS QRs are interconnected by randomly distributed ZnS particles, forming a matrix rather than a simple assembly. Figure 2c shows the ZnS growth around the CdSe/CdS QRs. Instead of growing into a uniform thick shell, the ZnS on the CdS surface grow with an irregular shape and act as a bridge to crosslink CdSe/CdS QRs. The QRs in the QRAs also show a more irregular surface compared to the pristine QRs, suggesting a slight reconstruction of the surface. The surface reconstruction could help to release the lattice stress and stabilize the ZnS growing on the surface. Figure 2d displays the HAADF-STEM element characterization of the selected line in the image. The result shows the high Zn proportion between CdS QRs, proving the growth of ZnS within the QRs. The result meets the observations shown in Figure 2c.

**Figure 3:**
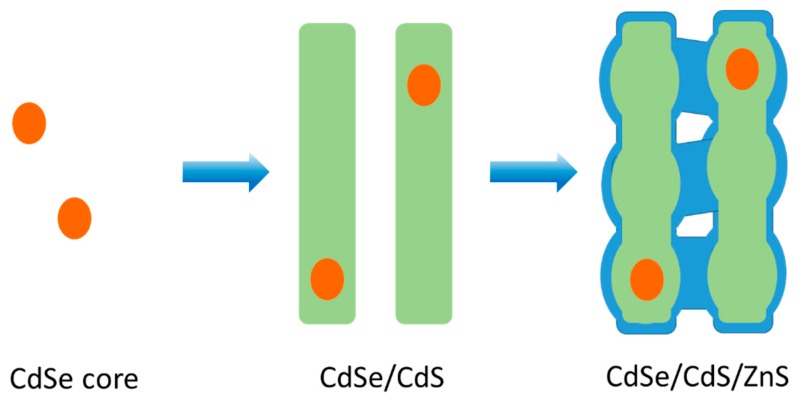
Schematic diagrams of the growth of CdSe/CdS/ZnS QRAs.

Based on the structure characterization, we suspect the reaction procedure occurs as illustrated in Figure 3. Zn(DTTC)_2_ slowly decomposes and firstly grows into a monolayer of ZnS on the surface of CdS QRs. With the continuous growth of ZnS, the CdS surface becomes unstable because of increased lattice stress, leading to slight surface reconstruction. During the process, some of the ZnS grows into large ZnS islands on the surface. Meanwhile, the highly concentrated CdSe/CdS tends to agglomerate due to the lack of surface ligands. Since the QRs are close to each other, the ZnS can contact the nearby QRs and finally form a ZnS particle bridge to connect the QRs. The interconnected QRs help to release the lattice stress, further inducing the continuous growth of ZnS into the QRAs structure.

### 3.2. Optical Characterizations of CdSe/CdS/ZnS QRAs

**Figure 4:**
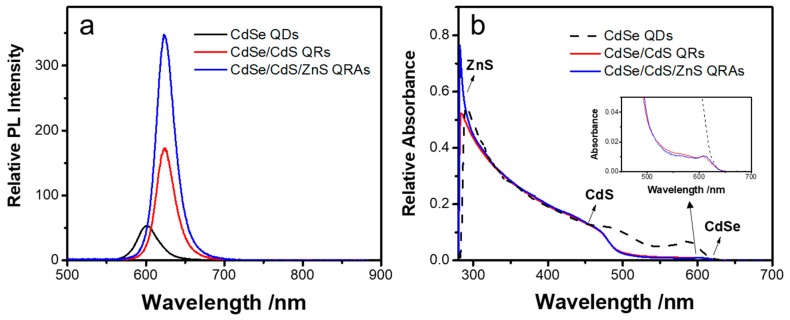
(**a**) Photoluminescence (PL) spectra and (**b**) UV-Vis absorption spectra of the CdSe QDs, CdSe/CdS QRs, and CdSe/CdS/ZnS QRAs.

Figure 4 depicts representative UV-Vis absorption and PL spectra from the prepared CdS QDs, CdSe/CdS QRs, and CdSe/CdS/ZnS QRAs. The UV absorbance of the samples was normalized at 450 nm in order to compare their absorption and emission features. The CdSe QDs emit at 600 nm with a full width at half maximum (FWHM) of 30 nm, as shown in Figure 4a. The growth of CdS on CdSe QDs induces a red shift of the PL peak to 623 nm and the peak width stays the same. The PL intensity of the CdSe/CdS QRs rises to about three times higher than the CdSe QDs with the same absorbance at 450 nm. Here, the CdSe/CdS QRs with a core size of 3.1 nm possess a type I or close to quasi-type II electronic structure [2,38]. The CdS shelling could passivate the surface traps on CdSe and help to confine the excitons, thus improving the quantum efficiency [39]. However, the carriers can still be easily quenched by the defects from the CdS surface. After the growth of the ZnS shell, the PL intensity further increases and the peak position remains unchanged, indicating a reduction in surface defects and improved confinement of the excitons.

As shown in Figure 4b, the first absorption peaks of the QRs and QRAs are located at 610 nm, representing the band-edge absorption of the CdSe core. Nevertheless, for pure CdSe QDs, the absorbance at 450 nm is only about 1.7 times that of the band-edge absorption of the CdSe QDs. The ratio rises to about 15 for the CdSe/CdS QRs and stays constant for the CdSe/CdS/ZnS QRAs. The QRs and QRAs display close absorption spectra in the visible region, and the absorbance of the QRAs rises significantly when the wavelength is below 380 nm. The results identify the growth of large volume CdS and ZnS. The absorption at the blue region is mainly attributed to the CdS shell. However, the ZnS has a wide bandgap that allows the penetration of blue light and only shows absorption at the UV region. The close absorption spectra of QRs and QRAs above 400 nm reveal no decomposition of CdS during the growth of ZnS. Therefore, the ZnS grows on the QRs by surface epitaxy growth rather than by ionic exchange between Zn and Cd cations.

**Figure 5:**
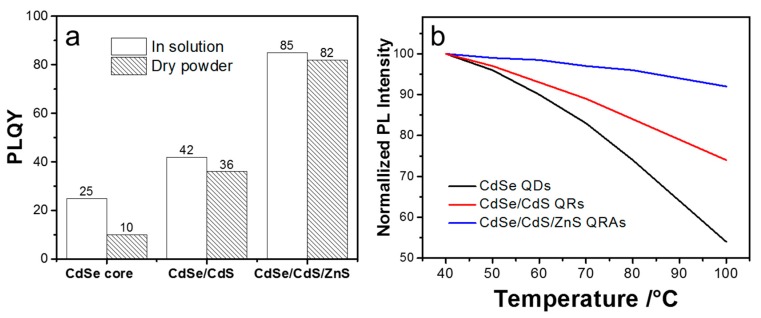
(**a**) Photoluminescence quantum yield (PLQY) of the CdSe QDs, CdSe/CdS QRs, and CdSe/CdS/ZnS QRAs in solution and in dry powder form; (**b**) fluorescent quenching of the materials at different temperatures.

Figure 5a shows the photoluminescence quantum yields (PLQYs) of the different materials in solution and as dry powder. The pristine CdSe QDs have a PLQY of 25% in solution but show strong fluorescent quenching after transferring into dry powder. This is caused by the near-field energy transfer and reabsorption due to the close distance among agglomerated QDs in powder form [40]. In comparison, the CdSe/CdS QRs present a higher PLQY of 42% in solution and a drop of 6% when being transferred into power, indicating that the large volume of CdS shelling effectively inhibits the reabsorption and reduces the fluorescent quenching by enlarging the average distance between CdSe cores and increasing the Stokes shift [41]. The QRAs in solution show significantly improved PLQY to a high level of 85%, and the PLQY drops by only 3% when transferring the QDs to powder. The results indicate that the ZnS encapsulation not only effectively reduces the surface traps and confines the excitons in the QRs, but also benefits the efficiency at a high concentration. Moreover, the QRAs gain further improved stability against thermal quenching. As shown in Figure 5b, the CdSe QDs display a strong temperature-dependent fluorescent quenching when excited at high temperatures. After CdS shelling, the quenching is largely reduced but still reaches close to 25% at 100 °C. Nevertheless, the QRAs preserve over 90% of the initial PL intensity when the temperature rises from 25 to 100 °C.

Based on the optical and thermal test results, we infer that the QRAs structure provides a more stable surface passivation for CdSe/CdS QRs compared to the thin layer of ZnS encapsulation. Firstly, the ZnS is a wide bandgap semiconductor. In addition to passivating the surface traps, the ZnS matrix can more effectively confine the excitons in CdSe/CdS QRs. As the result, the QRAs structure comprehensively improves the quantum efficiency and thermal stability of the CdSe/CdS QRs. In addition to the natural optical feature of the enlarged Stokes shift and strong absorbance at the blue light region from the core-in-rod structure, the stable ZnS encapsulation further provides the QRAs with high efficiency in power form without the obvious concentration and thermal quenching that are commonly observed in QDs. 

Table 2 compares the optical properties of different narrow emitting phosphors in WLEDs. It is known that the human eye has low visual sensitivity to wavelengths longer than 650 nm [22,42]. Hence, the spectrum over 650 nm will lead to considerable luminous loss for WLEDs. For this reason, the WLED application demands a narrow emitting phosphor in the red-light region. On the other hand, the display application also demands a narrow red emission peak to ensure high color purity and wide color gamut [13,23]. Commercial silicate or nitride-based red phosphors possess a broad emission peak that limits lighting and display applications. Recently developed fluoride-based phosphors such as K_2_SiF_6_:Mn^4+^ (KSF) provide a narrow emission peak at about 630 nm. However, they show a relatively low efficiency in the LED package and are sensitive to moisture. The fixed ultra-narrow emission peak of KSF is also more promising for display than WLED application. On the contrary, QDs and QRs have an adjustable emission wavelength and FWHM that benefit both display and lighting applications. The unique optical properties render the QRAs a more promising phosphor material in WLED application than “giant” CdSe/CdS/ZnS QDs.

**Figure 6:**
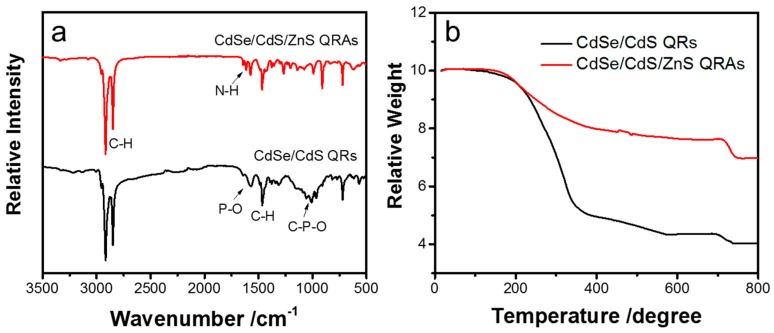
(**a**) FTIR spectra and (**b**) TG results of the CdSe/CdS QRs and CdSe/CdS/ZnS QRAs.

FTIR and TG characterizations were further employed to investigate the capping ligand on the surface of the CdSe/CdS/ZnS QRAs. The FTIR spectrum of CdSe/CdS QRs shows clear peaks at about 2780–3000 cm^−1^ and 1460 cm^−1^, which belong to the vibration of the C–H group (Figure 6a). The peaks at about 1000–1100 cm^−1^ and 1570 cm^−1^ can be assigned to the vibration of C–P–O group. Therefore, the surface of QRs is mainly capped by phosphonic acid ligands. For CdSe/CdS/ZnS QRAs, the fingerprint peaks of phosphonic acid present a relatively lower intensity. A new peak rises at about 1605 cm^−1^ that can be assigned to vibration of N–H in teh –NH_2_ group. Therefore, the CdSe/CdS/ZnS QRAs are modified with both phosphonic acid and amine. Moreover, the TG characterization shows a largely reduced proportion of organic ligands in QRAs compared with in QRs. As shown in Figure 6b, most of the organic molecules evaporate below 400 °C. The organic part accounts for over 50% of the total weight in QRs and its proportion declines to only 20% in QRAs. The results suggest that the organic surface ligands on QRs are largely replaced by inorganic ZnS during the formation of the QRAs structure. In this case, the QRs in the QRAs are mainly protected by ZnS instead of the original ligands. This further proves that ZnS plays a critical role in improving the efficiency and thermal stability of QRAs.

### 3.3. Photostability and LED Application of CdSe/CdS/ZnS QRAs

**Figure 7:**
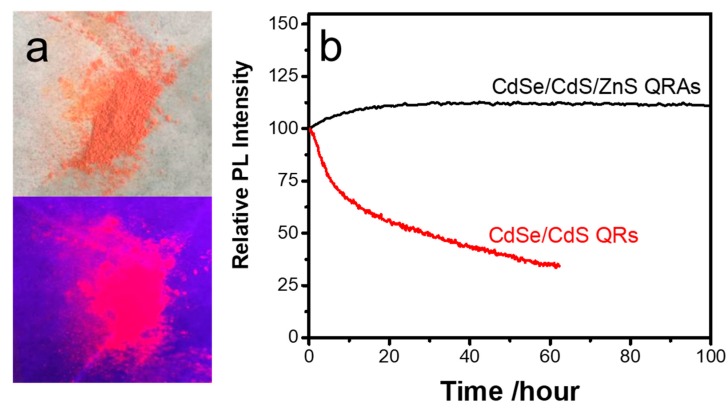
(**a**) QRAs powder under room light and UV irradiation; (**b**) long-term photostability of the CdSe/CdS and CdSe/CdS/ZnS QRAs under continuous excitation.

The QRs and QRAs were washed and dried into powder for the photostability test and the fabrication of LEDs. The dried QRAs powder displays a brick-red color under room light and bright red fluorescence under 365 nm UV light irradiation (Figure 7a). The dry powers of QRAs and QRs (about 50 mg) were collected and compressed into a thin film with a size of about 2 mm × 2 mm on a 1 cm × 1 cm alumina ceramic plate. The alumina plate was placed on a hotplate and the QRAs were excited by 440 nm laser irradiation at 0.1 W/mm^2^ to test the long-term photostability of the QRAs in air. As shown in Figure 7b, the QRAs became photochemically stable after an initial photoluminescence enhancement stage for approximately 20 h and presented slight fluorescent quenching for only 2% after continuous excitation for 100 h. In comparison, the QRs presented fast degradation, and the fluorescence intensity dropped to 50% after continuous excitation for less than 30 h. The results indicate the highly improved antioxidization property of the QRAs compared with that of the QRs.

**Figure 8:**
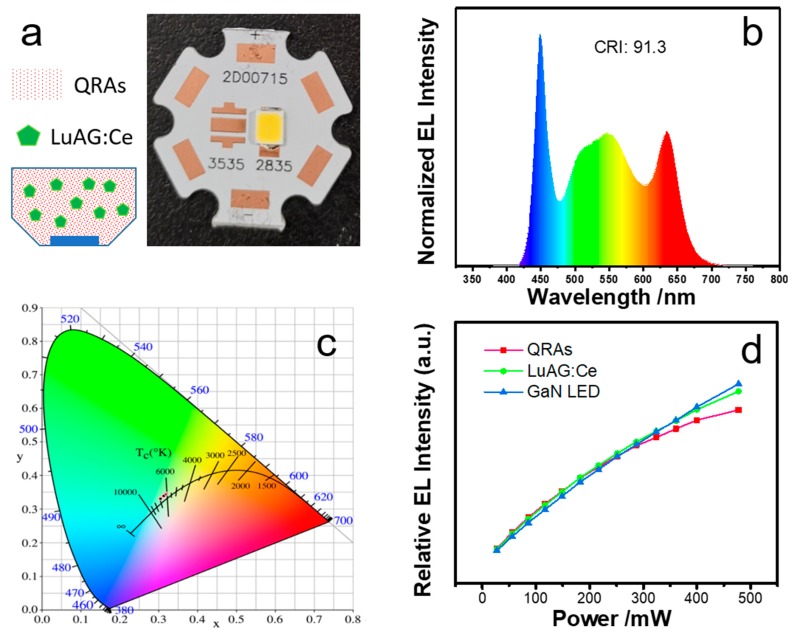
(**a**) The packaging structure and physical diagram of the QRA-based WLED package; (**b**) EL spectrum of the WLEDs; (**c**) color coordinates of the WLED at different driving powers; (**d**) relative light intensities of different light sources in WLED driven from 27 to 450 mW.

To further explore the optical characteristics of QRAs in practical applications, WLEDs were developed by using red emitting QRAs. Silica gel employing red-emitting QRAs and green-emitting LuAG:Ce was deposited and cured on top of a blue GaN chip to fabricate WLEDs. Figure 8a illustrates the LED structure and the fabricated WLED, and the electroluminescent (EL) spectrum of a WLED sample is shown in Figure 8b. The WLED with QRAs presents a luminous efficiency of 92.1 mL/W under 27 mW of driving power. The WLED has a high color rendering index (CRI) of 91.3 and a color coordinate at (0.3194, 0.3452), lying near the blackbody curve of the day light with a color temperature of 5000, as shown in Figure 8c. The curves in Figure 8d display the integrated red, green, and blue intensities of the WLEDs driven from 27 to 450 mW. The typical driving power for a medium power WLED is 350 mW. As shown in Figure 8d, the integrated light intensities of the three colors could maintain an approximately linear correlation to the operating power at up to 300 mW. Only when the power exceeds 350 mW, the red-light curve starts to show a clear downgrade trend, and the WLED presents a slight shift of the color coordinates toward blue. When the driving power is 477.4 mW, the color coordinate is (0.3048, 0.3303), as illustrated in Figure 8c. Therefore, the QRAs can meet the critical requirement for LEDs with on-chip type packaging. The test results reveal the high performance and promising potential of the QRAs in optoelectronic applications.

## 4. Conclusions

In summary, a facile synthetic approach has been developed for the growth of a CdSe/CdS/ZnS QRs-in-matrix structure. This method not only enables stable ZnS to be grown on the surface of CdSe/CdS QRs but also promotes the assembly of CdSe/CdS QRs with ZnS crosslinking. The unique QRAs structure provides thick ZnS encapsulation that effectively confines the excitons in the QRs and improves the antioxidization ability of the QRs. As a result, the QRAs present a high quantum efficiency up to 85%, thermal quenching of only 7% at 100 °C, and highly enhanced long-term photochemical stability. The stable ZnS encapsulation gives the QRAs high performance in practical LED applications. Moreover, the method has high potential to be applied to enhance optical performance and stability of the other non-spherical nanoparticles.

## Figures and Tables

**Table 1:** Material manufacturer and purity.

Materials	Suppliers	Purity
CdO	Alfa Aesar	99.5%
Se	Alfa Aesar	99.5%
Cd(St)_2_	Debo	98%
ODPA	EPSILON	98%
HPA	IRRITANT	98%
ODE	TCI	>90%
Zn(DDTC)_2_	TCI	>99%
HDA	TCI	>95%
TOP	TCI	85%
TOPO	Macklin	98%
Ethanol	Tianjin Damao	AR
Dimethylbenzene	Tianjin Damao	AR
Silicone-6662	Dow Corning	N/A

All chemicals were used directly without further purification unless otherwise stated.

**Table 2:** Optical properties of narrow emitting phosphors for white-light LED (WLED) application.

Materials	Peak Wavelength	FWHM	Efficiency as Power	Stokes Shift	Air- and Photostability	Thermal Quenching
KSF	~630 nm	~15 nm	60–70%	Large	Moderate	Moderate
QRs	~630 nm *	<50 nm *	<50%	Large	Unstable	High
QRAs	~630 nm *	<50 nm *	>80%	Large	Moderate	Moderate
Giant QDs	~630 nm *	<50 nm *	60–80%	Moderate	Moderate	Moderate

* Adjustable peak wavelength and FWHM.
